# Byron F. Coy

**Published:** 1881-08

**Authors:** 


### OBITUARY.

---

*Byron F. Coy, D. D. S.*—Died in Baltimore City, July 20th, 1881, Byron F. Coy, in the fifty-fifth year of his age. Dr. Coy was a native of Maine, but had resided in Baltimore for twenty-five years, occupying an enviable position in the dental profession, and having been successful in establishing a large and remunerative practice. Dr. Coy was a skillful operator and a gentleman of fine literary attainments. Prominent in the organization of the late Maryland Dental College, he held for several years a professorship in that institution, and was an honorary graduate of the Baltimore College of Dental Surgery.

Dr. Coy had been honored with the office of President of the American Dental Convention, and also of the Maryland and District of Columbia Dental Society. But mind is a brilliant not always set in iron, and the richest treasures of the intellect are often found in earthen vessels which a small force will suffice to shatter in pieces. Nature will not suspend her laws to favor those who violate them by injudicious burdens, even when heaped upon it by the purest motives. There are few things more calculated to stir our hearts with deep regret than to see one who has been a valuable member of a useful profession, and whose heart beats in sympathy with his fellows, descend into an untimely grave, where all that has been gained by many sacrifices is lost like the crystal waters which the thirsty traveler, in his haste overturns upon the desert sands.

The funeral services were held at Dr. Coy's late residence, No. 83 N. Charles Street, on Friday afternoon, July 22nd; the Rev. Dr. Smith officiating in the presence of a large attendance of friends and professional brethren. The interment was at Greenmount Cemetery, the pall bearers being Drs. R. B. Winder, H. H. Keech, R. Finley Hunt, E. P. Keech, F. J. S. Gorgas, and J. E. Scott.

At a meeting of the dental profession, held the same evening at the office of Dr. Chas. E. Duck, the following resolutions of respect and appreciation were adopted:

*Whereas* our beloved and respected brother, Byron F. Coy, has been torn from his sphere of usefulness by the inevitable hand of death, and recognizing to its fullest extent the large and valuable services which, in his distinguished position as pioneer and teacher, he has rendered in promoting the best interests of the profession, his liberality in imparting the truths wrung from his life of application and close observation, and his constant and untiring zeal in upholding a high standard of attainment and skill—

*Be it resolved*, That while we humbly bow in submission to the will of Almighty God, we deeply mourn his untimely end, and feel that our loss is a heavy one, and hard to bear.

*Be it further resolved*, That we hereby express our most heartfelt sympathy with his wife and orphan children, assuring them of our most earnest interest in their future welfare, and praying the merciful "God" to take them into His keeping, support them in this terrible affliction, and so bestow His blessings as to turn the house of mourning and grief into one of gladness and joy.

*Resolved*, That a copy of these proceedings be published in the Dental Journals, and one engrossed and forwarded to his widow.

